# The Migration of Hospitals

**Published:** 1903-03-28

**Authors:** 


### The Hospital.

A Journal of

The Medical Sciences and Hospital Administration.

Vol. XXXIII.—No. 861. Edited by Sir Henry Burdett, K.C.B., and Solomon C. Smith, M.D., M.R.C.P. [March 28, 1903.

### The Migration of Hospitals.

The meeting held last week at Grosvenor House to consider whether it would be possible, and, if possible, whether it would be desirable, to remove St. George's Hospital from its present site was animated throughout by a spirit of absolute reasonableness, of the best augury for the future of the institution. A committee with power to commence negotiations was appointed, and its report is likely, before long, to remove the questions at issue from the domain of speculation or conjecture into that of established fact. It may fairly be assumed that a good deal of what may be called the popular impression in favour of removal has been based upon the magnitude of the area now occupied by the hospital buildings, and upon the assumption that the whole of this area was available for the purpose of sale; whereas, as the meeting was informed, and as the majority of the governors probably knew before, the saleable area amounts only to about one-third of the whole. This third is the freehold property of the governors; while another third or thereabouts is held at a nominal rent from the Westminster estate under a lease which is perpetually renewable as long as the land is devoted to the purposes of a hospital, but no longer; and the remainder is held under a more modern lease from the same estate, practically under a similar condition. Hence it follows, not only that the governors can only dispose, for other than hospital purposes, of one-third of the area concerned, but also that any capitalist, or body of capitalists, desiring to purchase would either be limited to the third in question, or would have to deal separately with the Westminster estate for the attainment of any powers over the remainder. Instead, therefore, of the "million sterling," which has been mentioned in a non-medical contemporary as the sum which the governors might presumably realise from the site, it appears that the offer made by some unknown persons through a firm of agents amounted to considerably less than half that sum—to £350,000 or £375,000—and that it certainly would not suffice, except with the very strictest economy, for the bare accomplishment of the changes which are universally admitted to be desirable.

Sir William Bennett, whose admirable and conciliatory speech in proposing a resolution in favour of the principle of removal, if removal were possible, had a great influence upon the whole tone of the subsequent debate, had no difficulty in showing that the population served by the hospital had to a great extent departed from its vicinity, and that, as far as a large proportion of this population was concerned, a removal to a distance of a mile and a half or two miles from Hyde Park Corner into the crowded district of Fulham to the south-west would probably be of unmixed advantage. Such a removal, he might have added, would by no means be sufficient to restore the hospital to a position like that in which it was founded, in open fields far to the west of the London of that day, and might even result in financial ruin. The committee are not committed to this or any site, and there are far more suitable, better, and cheaper sites available. Mr. Warrington Haward pointed out that the present site, ideal as it might be considered for some purposes, was by no means ideal for a hospital. Bounded on three sides by busy thoroughfares, it was alike noisy and dusty; and noise and dust were alike prejudicial or even dangerous to the sick. Mr. Haward briefly disposed of the objections which had been raised to removal, on the score that it might be inconvenient to the members of the medical and surgical staff, by the simple observation that the members of the staff would do their duty. It appeared, however, that the question had been submitted for their consideration, and that, by a majority of 16 to 6, they had passed a resolution approving of removal if it could be carried out under the conditions which, on all sides, appear to be regarded as essential. Dr. Dickinson, who moved an amendment recommending the purchase of four houses in St. George's Place and the making of such other arrangements as might be practicable on the present site, which would mean the removal of the medical school, had no very convincing arguments to advance, and was even forced to fall back upon the suggestion that the present conspicuous position, if it did not produce annual subscriptions, was in all probability a source of legacies. He was seconded by Sir William Karslake, K.C., who had nothing to urge but a mild objection founded on an imaginary difficulty about the time of payment and the time of giving possession to the purchaser, a difficulty which was promptly disposed of by Sir Isambard Owen. After the acceptance, by the mover and seconder of the first resolution, of a verbal amendment suggested by Sir Henry Burdett, which rendered it somewhat less positive, and made the governors undertake to "consider," instead of to "consent to," any practicable proposal which a committee *ad hoc* might be able to bring before them, the resolution was carried by a large majority. The general effect of the proceedings is an acknowledgment by the governors that the hospital cannot be properly conducted in the present cramped area, that the means of extension must be found, and that they would be more completely furnished by removal than by any possible reconstruction on the present site. It is hardly possible that these views will fail to receive practical recognition at no distant period; and, in the meanwhile, it was understood that the purchase of the four houses in St. George's Place would be proceeded with.

